# A brief home-based palliative care learning experience for medical students and resident doctors in Okinawa, Japan

**DOI:** 10.1371/journal.pone.0218780

**Published:** 2019-06-25

**Authors:** Hiroaki Nagano, Haruo Obara, Yoshihiro Takayama

**Affiliations:** 1 Department of Respiratory Medicine, Okinawa Chubu Hospital, Okinawa, Japan; 2 Department of General Internal Medicine, Okinawa Chubu Hospital, Okinawa, Japan; 3 Department of Infectious Disease, Okinawa Chubu Hospital, Okinawa, Japan; King Saud University, SAUDI ARABIA

## Abstract

**Background:**

The clinical component of medical education for students and resident doctors in Japan occurs almost entirely in the hospital setting. Because of this inpatient focus, graduate medical education clinical training often fails to expose physicians-in-training to the challenges that patients may face in the outpatient or home setting.

This is a descriptive study in which we explore what participating students and resident doctors learned through our brief home-based teaching experience.

**Methods:**

From June 2016 to December 2017, attending physicians on the internal medicine service had medical students and resident doctors accompany them on home care visits. Participants were selected by convenience sampling based on their rotation availability. After the home visit and the interactive discussion, the participants were expected to prepare a reflective journal on their experience and learning. Thematic analysis was applied, and key themes were developed based on Kolb’s ELT (Experiential learning theory). Three months after completion of the experience, semi-structured interviews were individually conducted assessing participants’ self-perceived changes.

**Results:**

Thirty-two medical students(10) or residents(22)participated in a home visit. Thirty of these learners were able to complete a reflective journal. Using thematic analysis, we identified 2 domains and 6 key themes from the participants’ perceptions.

Participants recognized the importance of patient-centered care, inter-professional collaboration of the home care team, and reconceptualized the meaning of medical practice and their professional identity as a doctor.

Three month post-experience interviews were completed on 12 of the original 30 participants who completed the reflective journal. 2 domains and 6 key themes from the residents’ experiences and perceptions were generated. The participants reported an increased attention to the daily lives and social situations of their hospitalized patients, and an extension of their focus beyond the clinical medical treatment of the patient.

**Conclusion:**

The experience of a brief visit to a patient’s home is a novel educational approach that may potentially provide medical students and resident doctors with opportunities to learn about out-of-hospital, patient-centered, home-based medical care.

## Background

Even though nearly half of all patients state that they would choose to spend their final days of life at home, this often turns out to be difficult and most patients eventually die in the hospital [1][2]. It is important for medical staff to inquire about the lifestyle and backgrounds of dying end-of-life patients so that the doctors can deliver care that meets patients’ expectations. Previous studies have reported that through home-based teaching trainees learn how to place more emphasis on quality of life, focus on patient-centered care, and more deeply appreciate the interconnected experiences of the patient and family during the illness [3–16]. Home visit experiences may help medical staff understand the background of the patient's life and promote cooperation with other medical staff who may be involved in the patient’s care [3,9–12, 16–18]. However, in Japan, most clinical education programs for medical students and resident doctors are conducted within the hospital setting. Our residency program has similar problems. As a result, residents and students learn to approach the clinical practice of medicine primarily from the viewpoint of inpatient care. This approach may not expose them to the challenges that patients and their families face when they are forced to deal with health care issues at home.

In 2013, we established a home visit team to help fulfill patients’ wishes of dying at home. Since the home visit team was established, we wanted to take advantage of the opportunity and utilize these home visits for educational purposes. However, in our residency program, it was difficult to create a specific rotation for home visit care. Subsequently, we developed a unique program to take medical students and resident doctors along on a home care visits while they were assigned to other clinical rotations. We expected that this opportunity would allow young doctors and medical students to experience medical care rooted in the community and medical care that focused on patients’ lives through the home visit care.

This descriptive study explored the learning experiences of a group of medical students and residents working in a Japanese teaching hospital as a result of brief home-based palliative care visits.

## Methods

We employed a qualitative investigation to explore residents’ psychological and emotional learning after a home visit. An overview of the program is shown in Table 1.

**Table 1:** An overview of the program.

Aims of the program:	Promoting young doctors and medical students to recognize the importance of patient-centered care
Frequency	Once a week
Academic year	MS(6th year of study),PGY1, PGY2, PGY3, PGY4
Organization	Attending physician, education office of residency program
Patients	Advanced cancer patients, with an expected prognosis of approximately one month or less.
Participants’ activity	Performing the physical examination, taking part in the care conference, clinical record documentation.
Supervision	Three attending physicians (HN, YT and HO)
Assessment	Formative assessment by writing reflective journals

### Planning to introduce home visits and reflections

From June 2016 to December 2017, attending physicians on the internal medicine service had students and resident doctors accompany them on home visits. Our medical team was composed of medical doctors, dentists, and dental hygienists, including the main author (HN).

We used convenience sampling to select the participants for this study. The post-graduate year one (PGY1) resident doctors complete recurrent two-week rotations in each department section. When PGY1 residents were too busy to attend the home visit, we recruited medical students or post-graduate year 2 (PGY2) resident doctors or senior resident doctors (PGY3 or PGY4). Medical students were all in their 6^th^ year of study (final year) on hospital rotations. All the patients who received visits had advanced cancer, with an expected prognosis of approximately one month or less. In all cases before the home visit, we contacted the patients and their families for verbal approval to include resident and medical student participation. Home visits coincided when the need for such visits arose and medical students or residents accompanied us as their schedule allowed. The frequency of home visits was about once a week. Each home visit lasted 1 to 2 hours. On the way to the home visit, the attending physician reviewed the following with the participating medical student or resident physician: the patients’ clinical course, purpose and need for the home care visit, as well as the roles of the other health professionals comprising the home visit team. As part of the home visit, we gently inquired into the patient’s and family members daily life, functioning, coping and concerns, in addition to performing routine clinical care including prescription of medications. In the patient’s home, the participant assisted the attending physician with patient evaluation, took part in the care conference discussion with the family and documented the visit proceedings in the clinical record. The care conference format included: Explanation to the patient and family about the clinical situation including possibilities of what could happen in the future; asking the patient and family to express any concerns about physical and spiritual pain which they may be experiencing; and showing the support of the medical team. In the conference, the discussion was focused on the patient’s living will, improving the patient’s comfort level at home, and explaining the expected clinical course, including active dying.

As for the activities during the home visits, there was no difference between the medical students and resident doctors.

After returning to the hospital, we had an individual facilitated discussion with the participants. In the discussion, we queried participants about their reflections on what they experienced and felt. Attending physicians served as facilitators and were careful not to lead participants’ responses. The discussions were individually conducted in Japanese by the first author (HN) in a private room with each session lasting approximately 10 to 20 minutes. The main author listened and accepted the participants' opinions, and encouraged them to share their reflections. The semi-structured interview guide was based on Kolb’s Experiential learning theory (ELT) [19] and included the following questions:

- What part of the home visit experience left the biggest impression on you? What treatment/care was provided, and how did you feel when you saw the patient? (Experience/reflection)
- What lessons from this visit could be applied to future patient care? (Conceptualization/Planning)

After the discussion, we asked each participant to write a reflective journal based on their experiences and perceptions. Helen C. Richardson reported that reflective writing is said to encourage a writer to learn from an event, as it necessitates focused and analytical thinking [16][20]. The journals were collected and then translated into English. The translation was mainly performed by HN: the corresponding author of this study. HN had been directly involved with the participants and heard their raw impressions. HN read between the lines and tried to find hidden meaning in what was said or written. Two other authors who were familiar with English reviewed and analyzed both the Japanese and English journals. Thematic analysis based on Kolb’s ELT was independently performed by each author. Key themes were finally developed by consensus discussion of the three authors [19]. The Ethics Committee of Okinawa Chubu Hospital approved the study protocol. Our hospital Ethics Committee did not require informed consent, because the subject of this study was not patient-related but pertained to medical students or residents. Data utilized in this study were made anonymous and no direct quotes were attributable to participants.

### Post-visit interviews (3 month later)

Three months after the brief home visit, we performed semi-structured, individual follow-up interviews with participants who were still rotating at our hospital. The follow-up interview queried participants about their perceptions of the impact of the brief home visit experience on behavioral change in clinical practice or alterations in their concept about medical care. The aim of the follow-up interviews was to explore the continuous effect of participants’ learning effects as a program evaluation.

The semi-structured interview guide was also based on Kolb’s ELT [19]. The interview questions included the following:

- After the brief home visit experience, did you have any behavior changes in your hospital work? (Active Experimentation)
- As a result of the home visit, have you experienced any changes in thinking as a doctor? (Active Experimentation/Abstract Conceptualization)

Although the follow-up interviews were not recorded, the main author recorded notes from the interview. After transcribing the notes of the interview as field notes, thematic analysis was applied based on Kolb’s ELT [19], and key themes were developed by consensus discussion among the three authors.

**Fig 1:**
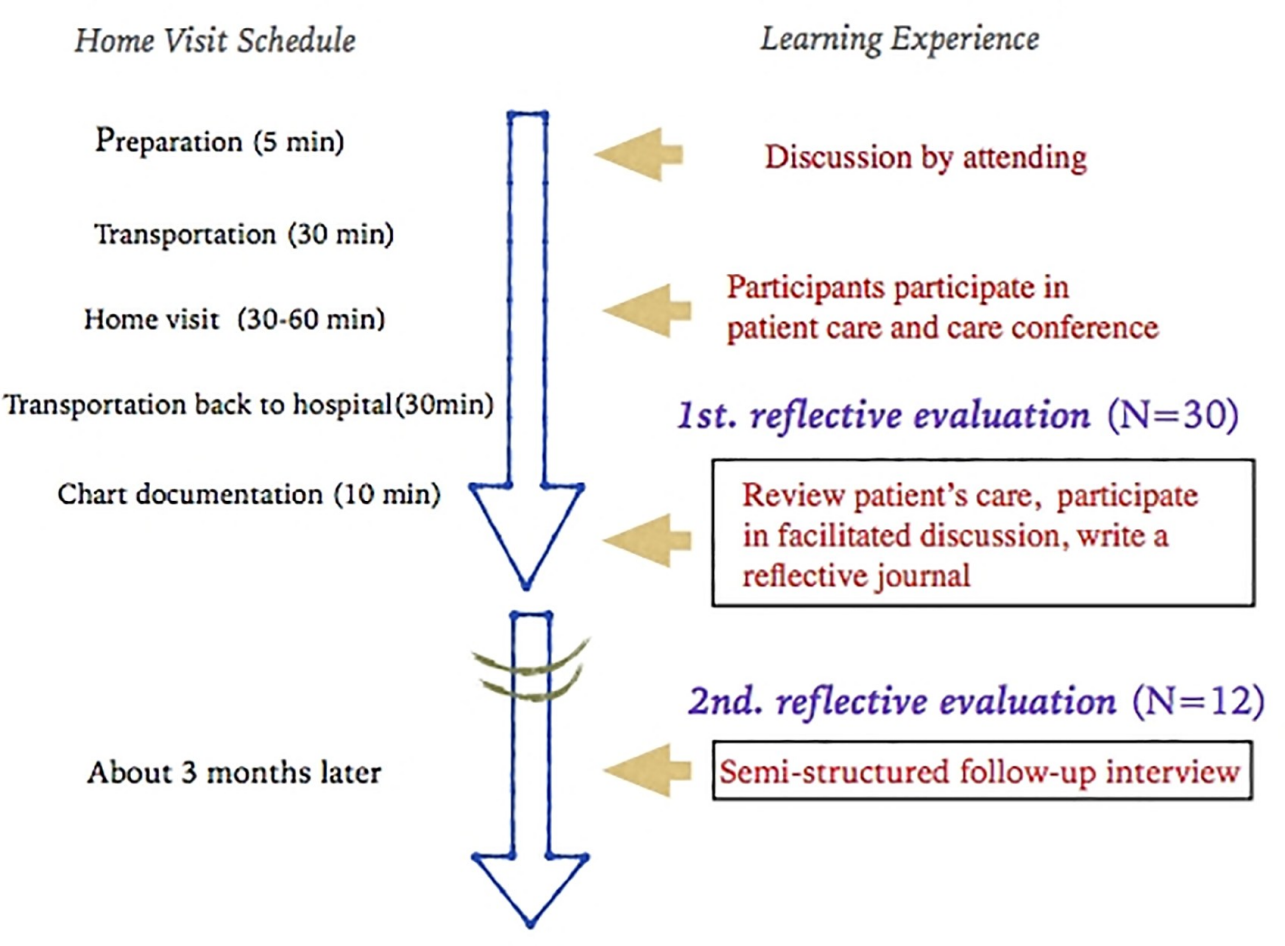
Flow chart for the brief home visit experience and reflection.

A flow chart illustrating the components of the home visit and reflection are shown in Fig 1

## Results

### Participant reflective journal

From June 2016 to December 2017, there were 32 participants who accompanied us on our home visits. We requested that all 32 participants complete a reflective journal, however two resident doctors (PGY-1) had to return to the wards for clinical duties immediately after the home visit and were unable to complete this task. The 30 participants who completed the journal included 10 6^th^ year medical students (33.3%), 12 PGY-1 resident doctors(40.0%), 4 PGY-2 resident doctors (13.3%), and 4 senior resident doctors PGY3 or PGY4 (13.3%). Using thematic analysis, we identified 2 domains and 6 key themes from the residents’ experiences and perceptions [Table 2**]**

**Table 2:** Participants’ perceptions: Key themes identified through the participants’ reflective journals after the home visit.

Theme (Kolb’s ELT [19])
I. Reflections on the experience of home visit (Reflective Observation) 1. The importance of emphasizing on quality of life, providing psychological and spiritual support at the end of life. 2. Broadening of perspectives from individual actions to inter-professional collaboration of the home care team 3. Renewed awareness of the difference between hospital-based medical treatment and home-based care II. Reconceptualizing the meaning of medicine and professional identity as a doctor (Abstract Conceptualization) 4. Reconsidering the role and goals of medical treatment at the end of life 5. Empathetic attitude towards patient's situation 6. Self-awareness of ideal image of the doctor

### I. Reflections on the experience of home visit

After the experience of home-based medical care, learner perceptions were mainly focused on patient-centered care. They appeared to develop an appreciation of the interconnected experiences of the patient and family during the illness as well as collaboration among the various types of medical staff involved in patient care.

1 The importance of emphasizing quality of life, providing psychological and spiritual support at the end of life.

*“I recognized that the home care medical staff concentrates on the patient-centered care*.*”*

*“I felt that the patient’s home had a calm atmosphere*, *a warm environment*, *and many smiles*.*”*

*“I was impressed because the doctors tried to listen to the patients’ pain and acknowledge the anxiety of patients and their families*.*”*

*“I felt that one of the important roles of a home visit was caring for the caregivers*.*”*

*“I realized that home visit care would facilitate the family’s acceptance of patients’ death*.*”*

2 Broadening of perspectives from individual actions to inter-professional collaboration of the home care team

*“Through the exposure to a home visit*, *I recognized the importance of multi-specialty collaboration*.*”*

*“In the care conference discussion during the home visit*, *I realized that doctors respected other care team members and closely cooperated with them”*

3 Renewed awareness of the difference between hospital-based medical treatment and home-based visit care

*“I felt that patients’ homes are usual spaces*, *whereas hospitals are unusual spaces*.*”*

*“I recognized that the hospitals where most resident doctors work are unusual places where the atmosphere is completely different from the patients*’ *living spaces”*

### II. Reconceptualizing the meaning of medicine, and professional identity as a doctor

Through exposure to patients’ personal lives, participants reconceptualized the goals of medical care at the end of life.

4 Reconsidering the role and goals of medical treatment at the end of life

*“Until the home visit*, *I had thought that medical treatment could strongly influence the patient's life*. *However*, *through the experience of a home visit*, *I realized that the most important element was the patient’s view of life including the relationship with society*, *people*, *ethics and living will*. *Medical intervention was only a part of the patient’s life*.*”*

*“I believed that medical treatments have to approach the patient’s mind and soul*.*”*

*“I realized that medical care is an act of pursuing happiness*.*”*

5 Empathetic attitude towards patient's situation

*“I noticed that I needed to see patients more politely because I found that patients came to our hospital from far away*.*”*

6 Self-awareness of ideal image of the doctor

*“I realized that I had to see patients as a “human” with dignity*, *not as a “sick person”*.

*“The home visit reminded me of the passion and goals I had when I wanted to become a doctor*.*”*

### Follow-up interviews (3 months later)

Approximately 3 months after the home visit, we conducted semi-structured, individual interviews with 12 participants as part of the follow-up evaluation. Eighteen participants were unable to complete the follow-up interview due to rotation schedule or moving to other hospitals. The 12 participants included 1 medical student (8.3%), 8 PGY-1 resident doctors (66.7%), 2 PGY-2 resident doctors (16.7%) and 1 senior resident doctor (8.3%). We analyzed the 12 field notes from the participant interviews. Using thematic analysis, 2 domains and 6 key themes from the residents’ experiences and perceptions were generated [Table 3].

**Table 3:** Conceptualization and behavior changes: Key themes analyzed from the field notes of semi-structured interviews 3 months after the brief home visit experience.

Theme (Kolb’s ELT)
I Rebuilding awareness for the patient’s care (Abstract Conceptualization) 1. The awareness of the importance of the patient’s lifestyle and background during inpatient care. 2. The awareness of the importance of advanced care planning II Active experimentation in daily clinical practice (Active Experimentation) 3. Providing the medical care that considers the patient’s values 4. Communicating with patients and families more closely and frequently 5. Respecting patients’ quality of life and wishes for care at the end of life (living will) 6. Paying more attention to the patient’s feelings and pain

### I. Rebuilding awareness for the patient’s care

After the brief home visit experience, some of the participants applied lessons from their home visit experience to their daily hospital work. The experience increased their awareness of their patients’ home life, which had previously been difficult to appreciate in the hospital setting.

1 The awareness of the importance of a patient’s lifestyle and background during inpatient care.

*“Through the brief experience of home visit care*, *I recognized that it was essential for us clinicians to learn the patients’ daily lifestyle and background*.*”*

2 The awareness of the importance of advanced care planning

*“I think that it is very important to discuss with the patient and their family about advanced care planning and I want to make efforts to encourage it in an early phase*.*”*

### II. Active experimentation in daily clinical practice

After the home care visit experience, some participants began to ask more about the patient's life and background, and began communicating with patients and families more closely. They also began to make efforts to respect patients’ dignity and way of living.

3 Providing medical care conscious of the patient lifestyle

*“I started to more pay attention to the patient’s lifestyle*, *nursing insurance and background when taking a history from them*.*“*

*“In order to maintain the lifestyle in their home*, *I started to encourage patients to do earlier referrals for occupational and physical therapy*.*”*

4 Communicating with patients and families more closely and frequently

*“I started to focus on the relationship between the patient and his/her family*.*”*

“*I felt more close to the patient’s family and started talking with them about everyday life”*

5 Respecting patients’ quality of life and wishes for care at the end of life (living will)

*“Through the brief experience of home visit*, *I was really impressed with the heartwarming attitude of team members to the patient and their family*. *Team members always respected the patient*’*s dignity and way of living*. *So I am making an effort to respect the patient’s way of thinking*, *and their living will*.*”*

6 Paying attention to the patient’s feelings and pain.

*“I began to pay attention to the patient's face*, *which shows their pain and feelings*.*”*

*“I began to ask the patients more frequently about what was what was worrying them most*.*”*

## Discussion

This qualitative study revealed Japanese medical students’ and residents’ perceptions, emotions and learning processes in response to a brief home visit exposure by applying Kolb’s ELT. Through the brief experience of our home visit, participants self-reported that they learned the importance of respecting the patient’s quality of life, providing psychological support, and inter-professional collaboration. They recognized the difference between hospital-based versus home-based care when managing palliative care patients. They also reconceptualized the meaning of medicine, and their professional identity as a doctor. Participants saw the attitude of our medical team, and gained lessons about a different approach to medical care. Three months after the home visits, although we did not objectively evaluate how their experience affected their actual practice, some of the participants reported an increased attention to the daily lives and social situations of their hospitalized patients, and that they had begun to focus beyond the medical treatment of patients. Recently Arai et al. have reported similar analysis methods and results [21]. Similar results have also been reported in other studies [3–5]. Although the results of our research are not significantly different in content from the previous studies, we have found that there are significant changes in awareness comparable to the previous research, but utilizing a short experience rather than a dedicated rotation. One of the most important and innovative features of our program is that we did not establish a set time or schedule during the rotation for home care visits. The home visits occurred as the need arose and medical students or residents accompanied us during their daily work on the hospital ward. The merit of this educational method was that participants did not have to sacrifice other important clinical training opportunities because we made use of unanticipated or unscheduled vacant time. Furthermore, by going to the patients’ homes as part of daily practice, hospital medicine and home care became seamless. We hope that resident doctors naturally come to think in terms of community-based health care through the experience of home care visits. Our teaching method is thought to be a new educational model that incorporates an intentional flexibility that makes it possible to provide residents with opportunities to learn within the context of extremely busy schedules and limited free time. Since this study was an exploration of learning using reflective journaling, we could not prove whether the same outcomes as the previous studies were obtained by this brief experience [3]. However, experiencing a home visit during the clinical rotation in teaching hospitals may improve the ability of medical students and residents to recognize the advantages and disadvantages of providing treatment in the hospital and at home in a well-balanced way.

This descriptive analysis of our program has some limitations. First, since the authors briefed the participants during travel, these could influence the views of the participants. However, we did attempt to explain both the merits and disadvantages of home visits to limit potential prejudice. Second, although our approach was not as rigorous a method of qualitative research than other approaches and we had a limited number of participants, the content of the discussion and the reflective journals were very rich and reached data saturation. Third, since pre- and post- test analyses were not conducted to evaluate the residents’ clinical ability, we were unable to establish objective causality of the effectiveness of the brief intervention. However, applying the Kolb’s ELT to the content of the reflective journals and post-intervention interviews, most participants seemed to express reflective observation (step 2) and abstract conceptualization (step 3). In addition, some of the participants seemed to reach the active experimentation (step 4) in the self-evaluation 3 months later [19].

There are also some areas in need of further exploration and improvement in our program. First, the optimal timing for the first experience of a home care visit in the course of a physician’s overall professional development is unclear. Although we were unable to establish this with our data, this is an area that would like to explore in future studies. If we can set the learning goals according to participant's educational level, then participating in a home visit regardless of their level of training should be useful. Second, we did not have a group discussion among participants to share their experiences. Since each brief visit experience was unique to each participant, it would be beneficial to share reflections with others as this is necessary to deepen the brief experiences in home care visit and lead to effective learning.

Although scheduling difficulties, lack of faculty time, and lack of resident time are barriers, these brief interventions may possibly improve medical students’ and physicians’ approaches to medical treatment and care at the end-of-life [22][23].

## Conclusion

The experience of a brief visit to a terminally ill patient’s home may not only be an effective way to help medical students and resident doctors understand the challenges that patients and their families face when illness strikes a household, but it may also have a positive influence that permanently changes how these young doctors think and practice. Our teaching method of including medical students and resident doctors on a home visit during their daily work rounds is an innovative educational model in that they were provided with the opportunity to learn patient-centered medical care by accompanying the attending physician on a home care visit. In addition, timely facilitated reflection may be necessary to deepen the brief experiences in home care visit and lead to effective learning. In the future, we hope this exposure to the challenges that patients face at home will help physicians in training be better prepared to provide compassionate patient-centered care, not only in the hospital, but also in the patient’s own homes.

## Abbreviations

PGY: post graduate year
ELT: Experiential learning theory
